# The Impact of the New Families Home Visiting Program on Depressive Symptoms Among Norwegian Fathers Postpartum: A Nonrandomized Controlled Study

**DOI:** 10.1177/15579883241255188

**Published:** 2024-07-30

**Authors:** Beate Solberg, Milada Hagen, Rigmor C. Berg, Kari Glavin, Malene Brekke, Kristin Marie Sæther, Anne-Martha Utne Øygarden, Nina Olsvold

**Affiliations:** 1 Centre of Diaconia and Professional Practice, VID Specialized University, Oslo, Norway; 2 Faculty of Health, VID Specialized University, Oslo, Norway; 3 Oslo Metropolitan University, Oslo, Norway; 4 Norwegian Institute of Public Health, Oslo, Norway; 5 University of Tromsø, Tromsø, Norway

**Keywords:** fathers, postpartum, home visits, depressive symptoms, mental health

## Abstract

Becoming a parent is a vulnerable life transition and may affect parents’ mental health. Depressive symptoms may occur in fathers, as well as mothers, during pregnancy and the postpartum period. The health service is expected to have a family perspective, aiming to support both parents. Despite this goal, mothers traditionally receive more support than fathers. Home visiting programs may provide enhanced guidance for new fathers and increased mental health support. The aim of this study was therefore to assess possible differences in level of depressive symptom in fathers receiving the New Families home visiting program compared with those receiving standard care from the Norwegian Child Health Service. A prospective nonrandomized controlled study with a parallel group design was performed. The Edinburg Postnatal Depression Scale (EPDS) was used to measure depressive symptoms in fathers (*N* = 197) at 28 weeks of their partners’ pregnancy (T1), at 6 weeks (T2), and 3 months postpartum (T3), in the intervention and the control group. The results indicate a prevalence of depressive symptoms (EPDS score ≥ 10) in Norwegian fathers of 3.1% at T1, 3.9% at T2, and 2.2% at T3 for the full sample. No significant EPDS score differences were found between the intervention and the control group at six weeks and three months postpartum. This suggests that the intervention had no clear impact on depressive symptoms during this time-period.

## Introduction

The transition to parenthood involves major life changes in roles, demands and expectations (Baldwin et al., 2019; Shorey & Chan, 2020). During this period, fathers may experience challenges and vulnerability related to their mental health (Darwin et al., 2017; Philpott et al., 2020). Depressive symptoms in fathers that occur during pregnancy and the first year after birth is often referred to as paternal postpartum depression (PPD) (Cameron et al., 2016; Paulson & Bazemore, 2010). Meta-analyses of international studies indicate a prevalence of PPD of 8.4% to 10.4%, with the highest rates identified 3 to 6 months after birth (Cameron et al., 2016; Paulson & Bazemore, 2010). PPD not only affects fathers’ health, but is identified to have a negative impact on parenting behavior, family relationships, and the health of mother and child, including the child’s risk of distress (O’Brien et al., 2017; Ramchandani et al., 2008; Ramchandani et al., 2011). Studies have reported that fathers often require support in the transition to parenthood (Hrybanova et al., 2019). While the Child Health Service (CHS) has traditionally been aimed at supporting the mother and child, today’s service providers are expected to have a family perspective that also includes the father (Norwegian Directorate of Health, 2017).

## Background

The Norwegian CHS is a part of the Norwegian health service at a municipal level. It is a voluntary, universal, free of charge service, used by 98% of all families (Norwegian Directorate of Health, 2017; Statistics Norway, 2021). The service is focused on health promotion and primary prevention, aimed at pregnancy, families with new-borns and children up to 5 years of age. The CHS offers a standard Child Health Program (CHP), which includes one home visit after birth and 13 subsequent clinical consultations at specific time points. They cover monitoring of the child’s growth and development, vaccinations and parental guidance and support. The Public Health Nurse (PHN) plays a key role in the service (Norwegian Directorate of Health, 2017).

A supplement to the standard CHP offered by the CHS is the New Families home visiting program (NF), which is a universal intervention, initiated and developed by the City of Oslo between 2013 and 2016 (Leirbakk et al., 2018, 2019). It offers the parents’ home visits from a PHN from late pregnancy until the child is 2 years old, in addition to the standard program.

The PHNs in the Norwegian CHS routinely meet almost all expectant and new parents (Statistics Norway, 2021), making it an arena for universal health care to couples in a vulnerable life transition. Relative to selective strategies, universal strategies are perceived as less stigmatizing, and more likely to be used (Fisher et al., 2018). Home visits are considered a good method to develop a relationship between the PHN and the parents in a safe environment, being more tailored to the parents’ need for support (Bäckström et al., 2021; Solberg et al., 2022). The mandate of both the Norwegian CHS and NF is to provide parental support, for both ongoing and new changes and challenges, including support for parents’ mental health (Norwegian Directorate of Health, 2017; Oslo Municipality, 2018).

This is recommended by the WHO standard of new-born care, which specify that parents should receive emotional support that is sensitive to their needs and aim to strengthen their capability (World Health Organization, 2022)

Traditionally, new mothers receive more support from health care professionals than fathers (Goldstein et al., 2020; Hrybanova et al., 2019). Men often hesitate to seek psychological help (Goldstein et al., 2020). If fathers are invited to home visits and visits at the CHS, this may increase their opportunity for receiving professional support (Solberg et al., 2022; Wells & Aronson, 2021). Research has identified that fathers appreciate home visits as a contribution to more tailored services and an arena for mental health support (Solberg et al., 2022). In addition, although fathers have reported less depressive symptoms when they receive professional support, both pre- and postnatally (Wells & Aronson, 2021), there is limited knowledge related to the effects of parental home-based support programs in the perinatal period (Minckas et al., 2023; O’Brien et al., 2017).

Controlled studies have reported that home visits can be an effective way to prevent, detect, and support postpartum depression in women (Milani et al., 2017), but there is a lack of knowledge about the impact of home visits and increased professional support on PPD. The aim of this study was to assess possible differences in the level of depressive symptoms between fathers receiving NF and those receiving standard care at the CHS. The main outcome was depressive symptoms assessed with the Edinburgh Postnatal Depression Scale (EPDS) and measured during their partners’ pregnancy, at 6 weeks, and 3 months postpartum.

## Methods

### Design

This is a prospective nonrandomized controlled study with a parallel group design. The study is a part of and used data from the New Families research project, which evaluate the experiences and impact of the NF home visiting program. The NF research project is registered on Clinicaltrial.gov (ClinicalTrial.gov identifier: NCT04162626).

We report in accordance with the Transparent Reporting of Evaluation with Nonrandomized Design (TREND) statement (Des Jarlais et al., 2004).

### Ethical Considerations

The study was conducted in accordance with the Helsinki Declaration (World Medical Association, 2013) and approved by the Regional Committees for Medical and Health Research Ethics in Norway (reference no: 2018/1378), and the Norwegian Agency for Shared Services in Education and Research (SIKT) (project number: 735207).

The participants received written and oral information about the study and its purpose. They were informed that all participation was voluntary and that they could withdraw at any time without consequences. The data were anonymized, treated confidentially, and stored in accordance with the Norwegian Personal Data and Health Research Acts using the Service for Sensitive Data platform (University of Oslo, 2016). Due to General Data Protection Regulations, we were not allowed to collect any information about the study’s nonparticipants (The Personal Data Act, 2018). The authors have no known conflict of interest to disclose.

### Participants and Recruitment

The NF study participants were recruited from five, of totally 15, city districts in Oslo, the largest city in Norway. The districts were selected by the municipality of Oslo, with the aim of ensuring the demographic and socio-economic representativeness of the population. Three districts were defined as intervention districts and two as control districts. Randomization of districts into intervention and control areas was not possible because the NF program had already been implemented in several districts and services when the research project started (described below).

In the intervention districts, the NF program was fully implemented, and the program had been running for at least two years. Each intervention district was carefully matched with a control district with the aim of similarity, in terms of population composition, sociocultural factors, birth statistics, immigrant proportion and work participation. The three intervention districts received the NF home visiting program in addition to the standard CHS program, while the control districts received the standard CHS program only (standard care). The participants’ allocation was determined based on their place of residence.

Pregnant first-time mothers, and the fathers of their expecting child, residing in the municipality of Oslo were invited to participate in the NF study. Expectant mothers were recruited by midwives or clinical secretaries when they attended antenatal consultations at the CHS. The women who expressed verbal interest in participating in the project were sent written information and a consent form by mail to return. The fathers were invited to participate through the mother’s involvement in the study and were sent a similar written, but separate, invitation and consent form. The inclusion criteria were being the father of the child of first-time pregnant women and living in one of the five districts in Oslo. Recruitment was from October 2018 to December 2019.

Based on a power analysis of depressive symptoms as measured with the EPDS with 0.80, an alpha level of .05 and an effect size of .5, we calculated a need for 64 participants in each of the two groups. To reduce the risk of losing power due to withdrawals, we set a goal of recruiting as many fathers as possible within the recruitment timeframe.

### Study Procedures

#### Description of the Intervention: The New Families Home Visiting Program

The NF home visiting program is a universal intervention initiated and developed by the City of Oslo between 2013 and 2016 (Leirbakk et al., 2018, 2019). It is offered as a supplement to the standard CHP and aims to strengthen the CHS’s health promotion and prevention work. Thus, it targets couples expecting their first child together, couples having their first child together in Norway and vulnerable multiparous parents. The program is universal and offers parents repeated home visits by a PHN from the 28th week of pregnancy until the child is two years old, while being individually tailored in terms of content and scope, with number of visits determined by each family’s needs. Both parents are encouraged to be present during the home visits, which last approximately 1 to 1.5 hours. The parents’ mental health is one of the recommended topics for the visits. Notably, NF is based on a salutogenic approach (Oslo Municipality, 2018) and aims to strengthen parenting skills and mobilize resources, focusing on change, motivation, and coping. It aims to establish early a supportive relationship between the PHN and the family, for best possible guidance. By using a primary nurse model, both home visits and the standard CHP are provided by the same PHN. The parents are provided with the direct mobile number of their PHN, giving them an opportunity for regular and direct contact (Oslo Municipality, 2018).

The PHN conducting home visits received training, which was organized by the CHS. The training workshops included descriptions of the NF program, theory such as salutogenic theory and the concept of self-efficacy, and guidance in conversational techniques, such as “Motivational interviewing” and “Empathic communication.” The PHNs training included mentored home visits and self-reflection. The theoretical foundation and implementation of the NF program are presented in a separate program manual (Oslo Municipality, 2018).

#### Description of the Control Group: Standard Child Health Program

**Figure 1.**
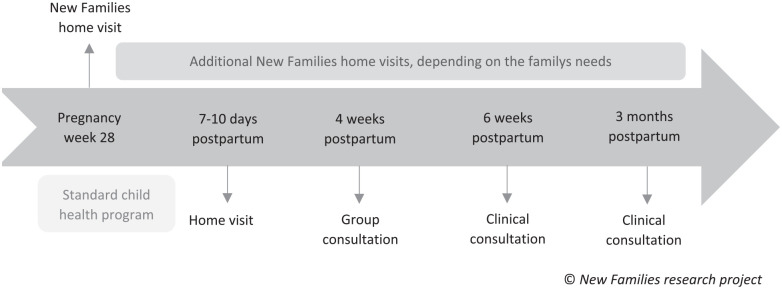
Timeline of the New Families Home Visiting Program in the Context of the Standard Child Health Program

The standard CHP is offered as a universal program, including one home visit after birth and 13 clinical consultations at specific time points from the child’s birth and up to 5 years of age, provided by a PHN. The content is regulated by national guidelines and cover monitoring of the child’s growth and development, vaccinations and parental guidance and support (Norwegian Directorate of Health, 2017). Until 3 months postpartum, the CHP provides one home visit 7 to 10 days postpartum and three clinical consultations. The timeline of the NF home visiting program in the context of the standard CHP is presented in Figure 1.

### Data Collection

Data were collected using self-reported questionnaires sent to the participating families by mail at the 28th week of pregnancy (T1), at 6 weeks (T2) and at 3 months (T3) postpartum. All questionnaires were sent to the families to be returned in pre-paid envelopes with advanced notification of distribution via Short Message Service (SMS). We sent up to two reminders by SMS if questionnaires were not returned.

All study information and questionnaires were available in nine different languages in addition to Norwegian (English, Arabic, Lithuania, Pashto, Polish, Somali, Tamil, Turkish, and Urdu). Available languages were chosen based on ethnicity in the five city districts. Data were collected between October 2018 and June 2020.

### Outcome Measure

The main outcome of the present evaluation was depressive symptoms in fathers as measured with EPDS (Cox et al., 1987). EPDS is a questionnaire originally developed to screen for depressive symptoms among postpartum women, but it is validated for use to assess fathers (Berg et al., 2022). The questionnaire consists of 10 self-report items addressing feelings experienced over the previous 7 days, using a 4-point Likert-type scale (0–3) with an overall score between 0 and 30. A higher score indicates more severe symptoms of depression (Cox et al., 1987).

Validation studies recommend different cut-off scores, depending on sample size, time of completion, cultural differences and gender (Berg et al., 2022). The originally suggested cut-off for women is ≥10 (Cox et al., 1987; Eberhard-Gran et al., 2001). Recommended cut-off scores for men differ, ranging from ≥5 to ≥13, with ≥10 as the most frequent (Berg et al., 2022).

EPDS has good internal consistency, with a reported Cronbach’s alpha coefficient ranging from .73 to .88 in pregnancy, and .60 to .88 at 0 to 6 months postpartum (Berg et al., 2022). In this study, the Cronbach’s alpha coefficient was .74 at T1, .80 at T2, and .75 at T3. Thus, we consider the instrument to have good internal validity in our sample.

### Statistical Analysis

Continuous variables are presented with median (minimum–maximum) and categorical variables as counts and percentages. To compare groups, we performed Mann–Whitney *U* test for continuous variables and chi-square test for categorical variables. We report continuity corrections regarding chi-square test for 2 × 2 tables, and Fishers’ Exact test two-sided *p*-values when small numbers (≤5 in any cell).

As the participants, were not randomized, we compared the groups at baseline (T1) with regards to age, nationality, number of children, marital status, education, family income, employment, and previous and present mental illness.

For the analysis of between-group differences in the outcome measure, a general linear model (GLM) for repeated measures was fitted. The model consisted of two covariates: measurement time and group (intervention/control). In addition, we constructed the interaction term Time × Group (intervention/control). This was entered in the model as a covariate to assess if changes in EPDS mean score over time were different in the intervention and control group. There were no statistically significant differences between the intervention and the control group in demographic related variables at baseline; therefore, no other covariates were included in the GLM.

Between-group differences were computed as the difference in change in the intervention group and the control group assessed from baseline to 6 weeks and baseline to 3 months. All estimates are presented with 95% confidence intervals (95% CI).

All analyses were conducted according to intention-to-treat principle (ITT), thus all participants in both groups were included irrespective of the number of home visits they had received from the NF intervention.

To assess possible selection bias, we tested for possible baseline differences between responders and nonresponders at T2 and T3 regarding age, education, and family income, performing Mann–Whitney U test or chi-square test, as appropriate.

Descriptive analyses were performed to determine the prevalence of EPDS ≥ 10 reported as counts and percentages. The results are presented as point estimates and raw number. Internal consistency and reliability were assessed by calculating Cronbach’s alpha for the total scale of EPDS. All statistical analyses were performed with the Statistical Package for the Social Science (SPSS), release 28 and Stata ver. 17. A statistician (MCS) was consulted for planning of the study; she provided supervision concerning the choice of statistical methods and participated in data analysis. All tests were two-sided. *p*-value < .05 was considered statistically significant.

The EPDS consists of 10 self-reported items. The proportions of responders with missing values at T1 were 0.9% for both the intervention group and the control group, at T2 0.2% for both groups, and at T3 0.1% for the intervention group and 0.2% for the control group. Based on the small number of missing values, these are handled as missing data and not imputed.

A sensitivity analysis was conducted for the outcome measure based on validation studies (Berg et al., 2022) to assess whether a different cut-off score (≥11 and ≥12) would affect the estimate of the prevalence of depressive symptoms in fathers postpartum.

## Results

### Sample Description

**Figure 2.**
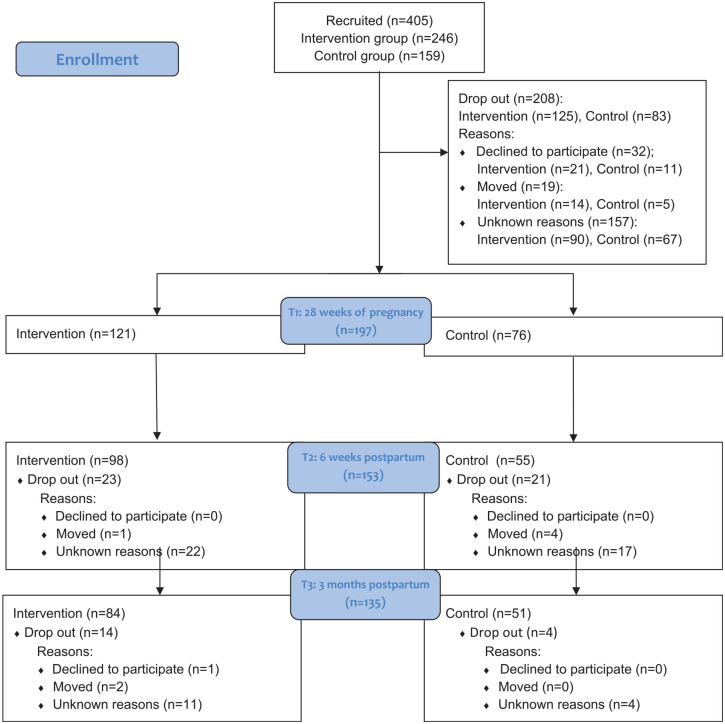
Flow Diagram of fathers at T1, T2 and T3, with reasons for dropouts

Of the 405 fathers invited to participate in the study, 197 were included (T1). The number of participants at each timepoint, the distribution between the intervention and the control group, along with the known reasons for drop-out, are presented in the flow diagram in Figure 2. The proportions of participants who dropped-out from T1 to T3 did not differ between the groups, with 30.6% drop-out in the intervention group and 32.9% in the control group.

The participants completed the questionnaires in three of nine available languages, where 187 (94.9%) fathers answered in Norwegian, 8 (4.1%) in English, and 2 (1.0%) in Arabic.

The intervention and control group did not differ statistically with respect to demographic data reported at baseline (age, nationality, number of children, marital status, education and family income, employment), as described in Table 1. When comparing responders and nonresponders at T2 and T3 with baseline, our data did not reveal any statistically significant differences between responders and dropouts regarding demographic variables.

**Table 1.** Sample Characteristics, Self-Reported Measures at Baseline

Characteristics of the participants				Participants (*N* = 197)						
	Total (*N* = 197)			Intervention group (*n* = 121)			Control group (*n* = 76)			Comparison
	*n*	%	Median (min-max)	*n*	%	Median (min-max)	*n*	%	Median (min-max)	*p*-value*
**Age**	195		32 (22–50)			32 (22–50)			32 (23–46)	0.87
Missing	2	1.0		1	0.8		1	1.3		
**Nationality**										0.27
Norway and the Nordic counties	168	85.3		100	82.7		68	89.5		
Other countries	28	14.2		20	16.5		8	10.5		
Missing	1	0.5		1	0.8		0	0.0		
**Number of children**										0.35
Primiparous	181	91.9		113	93.4		68	89.5		
Multiparous fathers	15	7.6		7	5.8		8	10.5		
Missing	1	0.5		1	0.8		0	0.0		
**Marital status**										NA
In a relationship	1	0.5		0	0.0		1	1.3		
Married/cohabiting	195	99.0		120	99.2		75	98.7		
Missing	1	0.5		1	0.8		0	0.0		
**Education**										0.60
Elementary/high school	34	17.2		20	16.5		14	18.4		
College/University < 4 y	63	32.0		36	29.8		27	35.5		
College/University > 4 y	99	50.3		64	52.9		35	46.1		
Missing	1	0.5		1	0.8		0	0.0		
**Family income, before tax, NOK**										0.62
<750 000	30	15.2		20	16.5		10	13.2		
>750 000	162	82.3		97	80.2		65	85.5		
Missing	5	2.5		4	3.3		1	1.3		
**Employment**										0.41
Employed	189	96.0		114	94.2		75	98.7		
Unemployed	6	3.0		5	4.1		1	1.3		
Missing	2	1.0		2	1.7		0	0.0		
**Previous mental illness**										0.09
No	186	94.4		111	91.7		75	98.7		
Yes	10	5.1		9	7.5		1	1.3		
Missing	1	0.5		1	0.8		0	0.0		
**Present mental illness**										0.68
No	190	96.5		117	96.7		73	96.1		
Yes	6	3.0		3	2.5		3	3.9		
Missing	1	0.5		1	0.8		0	0.0		

* Comparison: We report *p*-values as continuity correction for 2X2 tables and as Fisher’s exact test if counts in any cells were less than 5.

The majority of the fathers in the study were Norwegian or from a Nordic country (168, 85.3%), 162 (82.2%) were educated at a college/university level, 195 (99%) reported being married/cohabiting, 189 (96%) were employed, and 163 (82.2%) had a family income >750 000 NOK. Median age at enrollment was 32 years (range 22–50 years). Almost all, 181 (91.9%), were first-time fathers.

### Depressive Symptoms

Very few fathers in our study sample reported depressive symptoms. In the intervention group, depressive symptoms (≥10 on EPDS) were reported in 3 (2.5%) of the fathers at T1, 4 (4.1%) at T2%, and 2 (2.4%) at T3. These rates were similar in the control group, with 3 (3.9%) at T1, 2 (3.6%) at T2%, and 1 (2.0%) at T3. In total, the numbers indicate a low prevalence at all time points in both groups, with the lowest rate 3 months postpartum, as described in Table 2.

**Table 2.** Prevalence of Depressive Symptoms Measured by EPDS ≥10

	Total sample			Intervention group			Control group		
	*n*	%	[95% CI]	*n*	%	[95 % CI]	*n*	%	[95 % CI]
**T1: Pregnancy**	**6/197**	**3.1**	**1.1–6.6**	**3/121**	**2.5**	**0.5–7.1**	**3/76**	**3.9**	**0.8–11.4**
**T2: 6 weeks postpartum**	**6/153**	**3.9**	**1.4–8.3**	**4/98**	**4.1**	**1.1**–**10.1**	**2/55**	**3.6**	**0.4–12.3**
**T3: 3 months postpartum**	**3/135**	**2.2**	**0.4–6.3**	**2/84**	**2.4**	**0.3–8.4**	**1/51**	**2.0**	**0.0–10.1**

In sensitivity analyses, see Table 3, cut-off values were set at ≥11, and ≥12 (Berg et al., 2022). This resulted in rates of depressive symptoms being 1.5%, and 1.0%, respectively for the total sample at T1, 3.9% and 3.0% at T2, and 1.3% and 1.5% at T3. Thus, our sensitivity analyses showed that irrespective of cut-offs applied, there are few fathers with depressive symptoms, a slight increase from T1 to T2 and a decrease toward T3.

**Table 3.** Sensitivity Analyses EPDS

Alternative Cut-off score EPDS	*N* (%) ≥10	≥11	≥12
**T1: Pregnancy**			
Intervention group	3 (2.5%)	2 (1.7%)	2 (1.7%)
Control group	3 (3.9%)	1 (1.3%)	0 (0.0%)
T1 total	6 (3.1%)	3 (1,5 %)	2 (1.0%)
**T2: 6 weeks**			
Intervention group	4 (4.1%)	4 (4.1%)	3 (3.1%)
Control group	2 (3.6%)	2 (3.6%)	1 (1.8%)
T2 total	6 (3.9%)	6 (3.9%)	4 (3%)
**T3: 3 Months**			
Intervention group	2 (2.4%)	1 (1.2%)	1 (1.2%)
Control group	1 (2.0%)	1 (2.0%)	1 (2.0%)
T3 total	3 (2.2%)	2 (1.3%)	2 (1.5%)

In total, 10 fathers (5.1%) reported having a history of previous mental illness, and 6 (3.0%) stated presently suffering from mental illness at T1. Among the fathers with a previous mental illness, one scored ≥10 on EPDS at all timepoints, while for the six fathers who reported having a present mental illness, five of them scored ≥10 at T2, and all at T3. This indicates an association between present mental illness and depressive symptoms.

### Between-Group Differences

There were no statistically significant differences in between-group changes of estimated marginal means in depressive symptoms (EPDS score ≥10) between the intervention group receiving NF and the control group receiving standard care from baseline to T2 (six weeks) or T3 (three months postpartum). The difference between the groups at T1 was 0.31 (–0.55, 1.17), 0.22 (–0.73, 1.16) at T2, and 0.08 (0.08, 1.15) at T3. The differences are described in Table 4.

**Table 4.** Estimated Marginal Means EPDS Score

	Intervention group		Control group		Between-Group differences		
	Mean	[95% CI]	Mean	[95% CI]	Mean	[95% CI]	*p*-value
T1 Pregnancy	3.31	2.78**–**3.84	3.00	2.34**–**3.66	0.31	–0.55, 1.17	0.48
T2 6 weeks	3.28	2.64**–**3.91	3.07	2.24**–**3.90	0.22	–0.73, 1.16	0.65
T3 3 months	3.11	2.54**–**3.68	3.02	2.29**–**3.76	0.08	0.88, 1.15	0.87

### Program Use

Almost one-third of the included fathers in the intervention group reported not receiving any additional home visits. In the families who received home visits, 66 fathers (77.6%) reported being present at the visit.

## Discussion

### Impact of the Program

The aim of this study was to assess the impact of the NF home visiting program on self-reported depressive symptoms among fathers postpartum. The data indicate no statistically significant differences in EPDS score between the intervention and the control group at 6 weeks and 3 months after birth.

The NF program is universal, with a comprehensive offer of parental support. However, the program does not specifically include depression support, but rather focuses on general support, including mental health. This may have influenced the program’s lack of impact on PPD. Universal approaches supporting fathers have a clear value in general (Bäckström et al., 2021), but regarding paternal perinatal mental health challenges, there seems to be a need for more targeted interventions (Raminov et al., 2016).

It is suggested that when evaluating interventions targeting father’s mental health, a wider range of mental health outcomes should be considered (Raminov et al., 2016). In a systematic review, only one of five interventions with the aim to reduce and prevent PPD showed a significant reduction in PPD (Raminov et al., 2016). Even if studies indicate an association between home visit interventions and better mental health in fathers (Burcher et al., 2021), research highlights the need for more comprehensive and validated instruments when evaluating pre- and postnatal care, focusing more on measuring parents’ experiences and satisfaction (Minckas et al., 2023). Further research with a qualitative design, such as in-depth interviews, may supplement intervention studies by giving more insight into fathers’ experiences with, in this case, the NF program and its impact on depressive symptoms.

The measurement time in this study was 6 weeks and 3 months after birth, the period when the prevalence of PPD in fathers generally is low (Cameron et al., 2016). Most fathers develop PPD between 3 and 6 months and up to 1 year after birth (Cameron et al., 2016), which means that the NF intervention might have had an impact if measurements were taken later in the first year postpartum.

The NF intervention is not standardized but tailored to the parents’ needs. Only 70.2% of the fathers reported that the family received NF home visits during pregnancy, and 77.6% of them were present at this visit. These findings indicate that the intervention did not reach all the parents or fathers. Studies suggest that fathers are not always informed or invited to participate during home visits (Høgmo et al., 2021), and, therefore, might need a specific invitation to attend home visits and the CHS (Wells et al., 2023). Early service use might influence their further engagement with the service and thus increase their opportunity for receiving support (Finlayson et al., 2023). It is known that PHNs are more aware of and supportive when it comes to mental health issues in new mothers, compared with fathers (Wells et al., 2017), and fathers often feel side-lined and unimportant (Leahy-Warren et al., 2023). Our results question to what degree the fathers were invited to the NF home visits, and if the program was adequately supportive of fathers’ mental health.

The results should be interpreted with regard to possible biases of the sample. The majority of participants had a high level of education, were in stable relationships, had good financial status and were actively employed. Many support programs target “high risk parents.” The NF program has a universal approach which includes apparently well-functioning parents (Leirbakk et al., 2018). That we did not find a statistically significant difference in EPDS score between the intervention and the control group might therefore indicate that the standard CHS program is satisfactory for our study population, as regards mental health support for depressive symptoms in the early postpartum period. However, NF enable PHNs to identify support needs among all parents, reduce stigma around visits, and deliver services at a level proportionate to the parents’ actual needs (Solberg et al., 2022)

### Depressive Symptoms

The primary outcome in this study was depressive symptoms as measured with EPDS among fathers in pregnancy, six weeks, and three months postpartum. Relative to many other studies, we found a lower rate of PPD in our sample. In meta-analyses the rate of depression in fathers from pregnancy until one year postpartum is reported to be 8.4% to 10.4% (Cameron et al., 2016; Paulson & Bazemore, 2010). Notably, the meta-estimates include studies from countries on five continents, with the largest number of studies conducted in the United States of America and Asia. North American studies report higher levels of depression in general, while European report the lowest. The studies used different measurement tools (Cameron et al., 2016). Rao’s et al. (2020) meta-analysis lends support to the low prevalence of PPD in European fathers, 5.52%, based on EPDS measures.

Compared with studies on PPD conducted in a European context, not targeting “high-risk parents” and measuring PPD within the same timeframes as us by self-reported EPDS with cut off ≥10 to 12, the rates of PPD are similar to our findings. In the total study sample, we found depressive symptoms during pregnancy in 3.1% of the fathers, while a comparable study with a similar sample from the United Kingdom identified the prevalence of PPD to be 3.9% (Ramchandani et al., 2008). In our study, 6 weeks postpartum, PPD increased to 3.9% and decreased to 2.2% 3 months after birth. Similar studies have reported prevalence rates between 3.6% to 5.0% 6 to 8 weeks after birth (Madsen & Juhl, 2007; Ramchandani et al., 2008), and 5.1% to 6.3% 3 months postpartum (Escriba-Agüir & Artazcoz, 2011; Massoudi et al., 2013).

The prevalence of depressive symptoms for the general Norwegian male population between the age of 20 to 49, is 10.2% to 11.6% (Krokstad et al., 2022). In light of the findings in our study, this may suggest that pregnancy and the first months postpartum, overall is a favorable time period for men with respect to depressive symptoms.

As previously mentioned, this study includes the period from pregnancy until 3 months postpartum. Studies report an increased rate of depressive symptoms in fathers 3 to 6 months after birth (Cameron et al., 2016; Paulson & Bazemore, 2010), and symptoms may even develop after the first year postpartum (Goodman, 2004; Kiviruusu et al., 2020). Lower rates have been observed during the second trimester and 0 to 3 months postpartum (Cameron et al., 2016). A systematic review and meta-analysis of studies validating EPDS in fathers identified the lowest range in EPDS score in fathers six-seven weeks postpartum (Shafian et al., 2022), Rao et al. (2020) found the lowest prevalence one to three months after birth, which supports our findings.

It is important to consider the results considering the screening tool used in the study. EPDS is developed for screening postnatal depressive symptoms in women (Cox et al., 1987). It has been validated for men postpartum (Edmondson et al., 2010; Loscalzo et al., 2015; Massoudi et al., 2013), but not in a Norwegian sample. Furthermore, no study has validated EPDS for men in the antenatal period (Berg et al., 2022). EPDS may be more sensitive in assessing female symptoms of depression. Men may be less expressive about their feelings and therefore score lower using a tool like EPDS (Shafian et al., 2022). Hence, further research should examine the content validity of EPDS used in fathers postpartum, to demonstrate the degree to which EPDS provides an adequate reflection of depressive symptoms in fathers.

Although EPDS is the most frequently used instrument for detecting depressive symptoms in men postpartum (Berg et al., 2022), there is a lack of agreement regarding cut-off for EPDS used in men, ranging from ≥5 to ≥11 in different studies (Berg et al., 2022). In this study we chose a cut-off ≥10. Our sensitivity analyses showed the results were robust with higher cut-off scores.

### Limitations

This study has limitations. We conducted a nonrandomized trial and selection bias is possible and difficult to assess with limited information about nonparticipants due to General Data Protection Regulations (The Personal Data Act, 2018). The participants were a highly educated, homogeneous group and thus the representativeness of our sample for the general Norwegian fathers’ population may be limited. Furthermore, depressive symptoms were self-reported and while EPDS is a valid instrument for measuring PPD, the instrument has not been validated for men in a Norwegian population of pre- and postpartum fathers. The study is limited to pregnancy and the first 3 months postpartum. The outcome measures may have been different if measurements had been taken later in the postpartum period. Studies have reported on several factors associated with PPD. Maternal perinatal depression has been found to be the strongest predictor for PPD (Goodman, 2004). This study does not include depression scores for maternal depression. It might have strengthened the study if prevalence of depression in mothers had been compared with the depression scores of fathers. Finally, the study may have been under-powered, with too few participants in the control group at T2 and T3.

## Conclusion

This study evaluated the impact of the NF home visiting program on self-reported depressive symptoms among fathers postpartum by examining differences in EPDS score between fathers receiving the NF home visiting program and fathers receiving the standard program from the CHS. We found no statistically significant differences in PPD between the intervention and the control group at 6 weeks and 3 months postpartum, indicating that the intervention had no clear impact on depressive symptoms during this time-period. In general, relative to the general male population, the prevalence of depressive symptoms in Norwegian fathers during pregnancy and the first 3 months postpartum appears low. This indicates that their partners’ pregnancy and the first months postpartum may be a favorable period for men with regards to depressive symptoms.

In general, health promotion interventions are often complex and difficult to evaluate. We recommend further research regarding mental health in fathers, with a wider range of outcome measures and supplementing quantitative studies with qualitative.

## Relevance for Clinical Practice

Paternal postpartum depression not only affects fathers’ health but also risk adversely affecting their parenting behavior, familial relationships, and the overall health of mother and child. Mental health in mothers and fathers are strongly correlated, and this calls for mental health support being given to both parents during pregnancy and the postpartum period. To meet this call a family perspective that includes fathers, in services provided by midwifes and public health nurses is needed. Research on the impact of home visits and increased professional support targeting fathers’ mental health is scarce. Therefore, this study seeks to contribute to new knowledge on the prevalence of depressive symptoms in fathers pre- and postnatally and the impact of increased support through a home visiting program in the primary health care service.
